# Effect of MgO Content in LF Refining Slag on Inclusion Removal and Cleanliness Improvement in GCr15 Bearing Steel

**DOI:** 10.3390/ma19020360

**Published:** 2026-01-16

**Authors:** Zhijie Guo, Yanhui Sun

**Affiliations:** Collaborative Innovation Center of Steel Technology, University of Science and Technology Beijing, Beijing 100083, China; d202210640@xs.ustb.edu.cn

**Keywords:** GCr15, Mg–Al binary inclusions, slags, spinel, LF refining

## Abstract

In this study, a laboratory-scale slag–steel reaction experiment was conducted to systematically evaluate the influence of the initial MgO content (3–7 wt.%) in LF refining slag on the cleanliness of GCr15 bearing steel. The assessment was performed from multiple perspectives by comparing the total oxygen content (T[O]) in molten steel, the inclusion area fraction, and the inclusion number density after 30 min of slag–steel interaction. To further elucidate the thermodynamic driving forces and kinetic mechanisms governing inclusion capture by slag, a predictive slag adsorption model was developed using an in-house computational code coupled with FactSage 8.1. Under conditions of slag basicity R (CaO/SiO_2_) ranging from 4.0 to 8.0, MgO content varying from 0 to 7 wt.%, and a constant Al_2_O_3_ content of 32 wt.%, the chemical driving force ΔC (the mass-fraction difference between slag components and inclusions), the slag viscosity η, and the combined parameter ΔC/η were calculated at 1600 °C for three representative inclusion types: Al_2_O_3_, MgO·Al_2_O_3_, and MgO. In addition, the model was employed to quantitatively characterize the adsorption capacity of slag toward Mg–Al binary inclusions under varying MgO levels. Both experimental observations and model calculations demonstrate that the slag–steel reaction markedly enhances inclusion removal, as evidenced by pronounced decreases in T[O], inclusion number density, and inclusion area fraction after reaction. With increasing MgO content in slag, T[O] and inclusion-related indices exhibit a consistent trend of first decreasing and then increasing, reaching minimum values at an MgO level of 5 wt.%. Further analysis reveals a positive correlation between the apparent inclusion-removal rate constant ko and ΔC/η corresponding to MgO·Al_2_O_3_ inclusions. Moreover, the slag’s adsorption capacity toward Mg–Al binary inclusions decreases overall as the MgO fraction in inclusions increases. Notably, when the MgO content in inclusions exceeds 29 wt.%, the adsorption capacity undergoes an abrupt drop, indicating a pronounced cliff-like attenuation behavior.

## 1. Introduction

Bearing steels play a pivotal load-bearing role in a wide range of critical industrial equipment, and their in-service performance directly governs the service life and operational reliability of related products. As a typical high-carbon chromium alloy steel, bearing steel can achieve high hardness, excellent wear resistance, and superior contact fatigue strength after appropriate heat treatment. Consequently, it is extensively employed in rolling-contact components with stringent reliability requirements, including those used in automobiles, rail transit systems, machine tool spindles, wind power transmission units, and aero-engines [1,2,3,4,5]. Among the various factors affecting the lifetime of bearing steels, non-metallic inclusions are widely recognized as one of the predominant initiation sites for premature flaking and rolling contact fatigue failure. Owing to the pronounced mismatch between inclusions and the steel matrix in hardness, thermal expansion coefficient, and plastic deformability, inclusions tend to induce local stress concentration under rolling contact stress fields. This facilitates crack initiation and propagation, promotes the formation of subsurface damage microstructures such as “butterfly wings”, and ultimately leads to a significant reduction in rolling contact fatigue life [6]. In particular, hard and brittle inclusions, including Al_2_O_3_, spinel (MgO·Al_2_O_3_), and TiN are more prone to serve as preferential nucleation sites for fatigue cracks due to their high hardness and limited toughness [7,8,9,10,11,12,13,14]. GCr15 is currently one of the most widely used high-carbon chromium bearing steels and has been produced on a large industrial scale with a well-established and stable manufacturing route, making it a representative product in steelmaking enterprises. However, this grade exhibits high sensitivity to non-metallic inclusions, and its fatigue performance is strongly constrained by the cleanliness level of molten steel. Therefore, from the standpoint of metallurgical process control, reducing the inclusion content and enhancing steel cleanliness is a critical pathway toward improving the service life and ensuring the performance stability of bearing steels [15,16].

Electric arc furnace (EAF) steelmaking uses arc energy as the primary heat source and is capable of efficiently recycling scrap steel while accommodating a certain proportion of hot metal addition. This feature underpins a flexible and high-efficiency short-process route. When combined with secondary refining operations such as ladle furnace (LF) treatment and vacuum degassing (VD), the EAF-based process can achieve deep desulfurization, deoxidation, and effective inclusion control, thereby markedly improving molten steel cleanliness. Owing to its high metallurgical controllability, compositional flexibility, and relatively low inclusion level, the EAF–LF–VD short-process route has become a major industrial pathway for producing high-quality bearing steels and various high value-added specialty steels [17,18,19,20,21]. In industrial bearing steel production, aluminum deoxidation is commonly employed. At the early stage of LF refining, dissolved oxygen in molten steel reacts with the deoxidizer Al, leading to the formation of a substantial amount of Al_2_O_3_ inclusions. As refining proceeds, the participation of MgO originating from refining slag and refractories further promotes the formation of magnesium aluminate spinel (MgO·Al_2_O_3_) inclusions in steel [22,23,24,25,26,27]. Magnesium aluminate spinel is one of the typical hard and brittle inclusion types in steels. It often exhibits irregular morphology and possesses a high melting point and hardness. Similarly to Al_2_O_3_, spinel inclusions are generally regarded as detrimental to steel service performance. For instance, during continuous casting, these solid inclusions tend to adhere to and accumulate on the inner wall of the submerged entry nozzle (SEN), which may cause nozzle clogging and consequently jeopardize flow stability and casting safety [28]. At present, two main strategies have been developed for controlling magnesium aluminate spinel inclusions. The first strategy involves Ca treatment to modify inclusions, transforming spinel into liquid or semi-liquid CaO–MgO–Al_2_O_3_ complex inclusions, thereby improving castability. However, such modified inclusions are prone to form large composite inclusions in the billet, increasing the risk of failure in ultrasonic inspection. Therefore, Ca treatment is generally unsuitable for bearing steels that require extremely high cleanliness levels [29,30]. The second strategy focuses on tailoring and refining slag composition to enhance the slag’s ability to absorb and remove spinel inclusions, which offers a more viable pathway for cleanliness improvement [31,32,33,34,35,36]. The removal of inclusions by slag absorption is typically described by three fundamental steps, denoted as R1, R2, and R3 [19,33,37,38]. Step R1 corresponds to the flotation of inclusions from the bulk molten steel to the steel–slag interface, which is primarily governed by inclusion type and size. Step R2 involves the transfer of inclusions across the steel–slag interface, and its driving force is strongly associated with the chemical driving force ΔC, defined as the mass-fraction difference between slag constituents and the inclusion phase. Step R3 refers to diffusion and mass transfer of inclusions after entering the slag, which is largely controlled by slag viscosity η. Numerous studies have demonstrated that the removal efficiency of magnesium aluminate spinel inclusions is positively correlated with the combined parameter ΔC/η, and that this parameter can be tuned through slag design [19,32,33,37,38,39]. Cunha Alves et al. [33] systematically investigated the effects of the chemical driving force ΔC and viscosity η of refining slag on the removal of CaO–Al_2_O_3_–SiO_2_–MgO inclusions, demonstrating that coupling ΔC and η (i.e., using ΔC/η) provides a more robust descriptor than analyzing either ΔC or η alone. They further proposed that the parameter could be optimized by controlling slag basicity and the C/A ratio. Guo et al. [19] reported that tuning ΔC/η effectively improves the removal of magnesium aluminate spinel inclusions and identified slag basicity and Al_2_O_3_ content as key variables governing ΔC/η. Wen et al. [38] evaluated the influence of CaF_2_ in refining slag on the removal behavior of MA inclusions in bearing steel and proposed an empirical relationship between the inclusion removal factor k_i_ and ΔC/η, expressed as ki = 2.681 × 10^−5^ × (ΔC/η) − 0.0035, where a higher k_i_ indicates more effective removal of MA inclusions.

In recent years, considerable progress has been made in elucidating the role of the slag parameter ΔC/η in governing inclusion removal behavior during secondary refining. Existing studies have primarily focused on how compositional variables such as slag basicity and the CaO/Al_2_O_3_ ratio regulate inclusion removal efficiency. Meanwhile, most researchers have considered that the addition of MgO primarily serves to reduce the corrosion of magnesia-based refractories by the slag. However, its influence on inclusion removal and the reasonable threshold of MgO content in slag have largely been neglected. In this study, by calculating the key parameter ΔC/η, the relationship between MgO content, steel cleanliness, and inclusion removal efficiency was clarified, and a reasonable threshold value was determined. Moreover, quantitative assessments of the slag adsorption capacity toward Mg–Al binary inclusions with varying MgO fractions remain relatively scarce, and a systematic understanding of the underlying trends has yet to be established. Motivated by these knowledge gaps, the present work combines laboratory-scale slag–steel reaction experiments with a predictive slag adsorption model developed using an in-house computational code coupled with FactSage 8.1. The model is employed to calculate the ΔC/η parameter under different slag compositions. On this basis, a comprehensive investigation is carried out to clarify the effects of the initial MgO content (3–7 wt.%) in LF refining slag on the cleanliness of bearing steel and on the slag’s adsorption capability toward Mg–Al binary inclusions. The findings are expected to provide theoretical guidance for refining slag design and for achieving more efficient inclusion removal in bearing steel production.

## 2. Experimental Section

### 2.1. Materials

The raw material used in this study was a GCr15 bearing steel billet produced in an industrial steel plant in China via the EAF–LF–VD–CC process route. Melting experiments were carried out in a 10 kg vacuum induction furnace. During melting, predetermined amounts of Al granules, FeS, and Fe_2_O_3_ powders were added to establish a molten steel composition representative of the LF refining stage. After completion of melting, the alloy was air-cooled, and the obtained ingot was employed as the master melt for subsequent experiments. To minimize contamination from exogenous impurities, the entire melting process was conducted in a high-purity MgO crucible. For slag preparation, CaO was introduced as a chemically pure reagent, while all other slag constituents were of analytical grade to ensure high raw material purity and experimental controllability. The powders were weighed according to the designed slag compositions to prepare 10 g slag batches, thoroughly homogenized, and then compacted using a hydraulic press. The mixed powders were pressed under a load of 15 MPa for 5 min, producing white cylindrical slag pellets with a diameter of approximately 25 mm, which were subsequently used in the slag–steel reaction experiments.

### 2.2. Procedure

**Figure 1:**
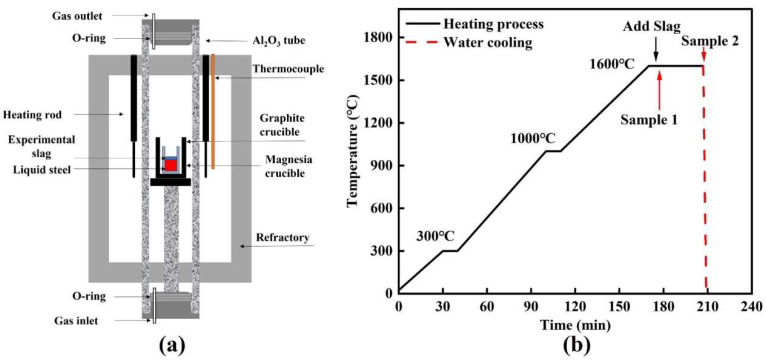
Experimental setup for high-temperature tests: (**a**) Si–Mo resistance furnace; (**b**) heating program.

Figure 1 presents the Si–Mo resistance furnace used in this study and the corresponding heating program. All high-temperature experiments were conducted in this furnace (Figure 1a). The steel specimens were initially extracted by mechanical machining (e.g., wire electrical discharge machining) and subsequently sectioned into small blocks. Prior to the experiments, the surface oxide scale was carefully removed by grinding to minimize interference from exogenous oxides. Approximately 150 g (±0.1 g) of the pretreated steel was weighed and charged into a high-purity MgO crucible (inner diameter: 38 mm; outer diameter: 46 mm). The MgO crucible containing the charge was then placed inside a larger graphite crucible (inner diameter: 50 mm) to provide mechanical support and additional protection. The assembled crucible set was subsequently introduced into the Si–Mo resistance furnace. To suppress secondary oxidation during heating, high-purity Ar gas was purged into the furnace for 10 min prior to temperature ramping to remove residual air and establish a stable inert atmosphere. The Ar purity was ≥99.99%, and the flow rate was maintained at 2.5 L·min^−1^. The furnace was then heated to 1600 °C according to the heating schedule shown in Figure 1b under continuous Ar protection. After reaching 1600 °C, the melt was held for 5 min to ensure sufficient homogenization. After the holding period, a pre-pressed slag pellet wrapped with approximately 3 g of high-purity iron foil (purity ≥ 99.99%) was added into the molten steel. Once the slag had completely melted, the first steel sample (Sample 1) was collected. Immediately thereafter, the entire crucible assembly containing the steel and slag was rapidly withdrawn from the furnace and water-quenched; this moment was defined as the zero time of the slag–steel reaction. The reaction was then allowed to proceed for 30 min, after which the second sample (Sample 2) was extracted following the same procedure and rapidly water-quenched. In industrial production, a slag–steel reaction time of 30 min corresponds approximately to 35–40 min of LF refining. At this stage, LF refining enters the middle-to-late period, during which MgO–Al_2_O_3_–based inclusions are dominant. This condition is highly consistent with the focus of the present study and also reflects actual industrial refining conditions. Therefore, the slag–steel reaction time was limited to 30 min in this work.

Table 1 summarizes the chemical composition of the molten steel at the zero time of the reaction. Unless otherwise specified, all compositions reported in this study are expressed in mass fraction (wt.%). Table 2 lists the designed initial compositions of the refining slags. The slag system employed was a quaternary CaO–SiO_2_–MgO–Al_2_O_3_ system. In the experiments, the slag basicity (CaO/SiO_2_) was fixed at 6 and the Al_2_O_3_ content was maintained at 32 wt.%. On this basis, the MgO content was systematically increased from 3 to 7 wt.% to investigate the effect of the initial MgO level on inclusion removal behavior and molten steel cleanliness. In addition, the saturated MgO content of the initial slags at 1600 °C was calculated using FactSage 8.1 with the FToxid database, and the results are also provided in Table 2. As shown in Table 2, when the slag MgO content reached 7 wt.% (corresponding to M5), it exceeded the calculated saturation limit at 1600 °C (6.7 wt.%). Therefore, M5 was identified as an MgO-saturated slag. In contrast, the MgO contents of slags M1–M4 were below the saturation level and were thus classified as MgO-unsaturated slags.

### 2.3. Analysis

The total oxygen content (T[O]) in molten steel was determined using an O/N analyzer (TCH600, LECO, USA), while the sulfur content (S) was measured with a C/S analyzer (EMIA-920V2, HORIBA, Japan). Trace elements in steel, including Ca, Al, and Mg, were quantified by inductively coupled plasma atomic emission spectroscopy (ICP–AES), with a detection limit of 5 ppm. To systematically characterize the size distribution, number density, chemical composition, and elemental distribution of non-metallic inclusions, statistical analyses were performed using an automated inclusion scanning system (Particle X, Phenom XL, The Netherlands). The scanned area for each measurement was approximately 30 mm^2^, and the minimum detectable inclusion size was 1 μm. The Particle X automated inclusion scanning analysis system is a software specifically designed for the statistical analysis of inclusions in steel, which has been reported in many published studies [18,19,38]. In the present study, more than 1000 inclusions were analyzed for each sample. The efficiency and workload of this automated analysis are far superior to those of manually searching for inclusions under SEM–EDS. For representative inclusion types, further examinations of morphology, characteristic size, and chemical composition were conducted using a scanning electron microscope equipped with an energy-dispersive spectroscopy system (SEM–EDS, Phenom XL, The Netherlands).

## 3. Results

### 3.1. Effect of Slag–Steel Reaction on the Chemical Composition of Steel

**Figure 2:**
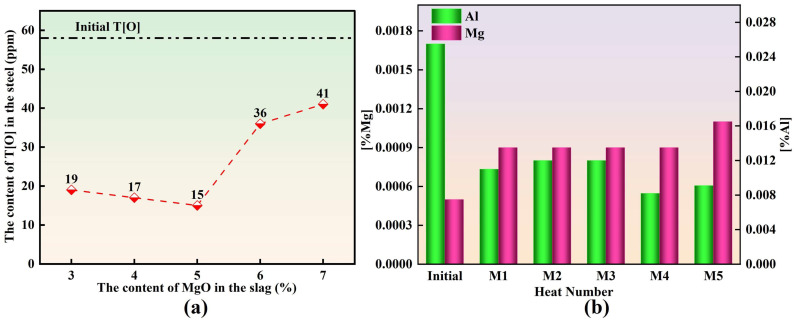
Chemical Composition of Experimental Steel: (**a**) T[O]; (**b**) T[Al] and T[Mg].

Unless otherwise specified, the concentrations of major elements in molten steel are reported as mass fractions, where 1 ppm corresponds to 1 × 10^−6^. Figure 2 illustrates the variations in T[Mg], T[Al], and T[O] during the slag–steel reaction. As shown in Figure 2a, the initial total oxygen content T[O] at 0 min was approximately 58 ppm. After 30 min of reaction, T[O] decreased markedly in all experimental groups. With the slag MgO content increasing from 3 to 7 wt.%, the T[O] value measured after 30 min exhibited a distinct “decrease–increase” trend, with corresponding values of 19, 17, 15, 36, and 41 ppm, respectively. The minimum T[O] was obtained when the slag contained 5 wt.% MgO. Figure 2b shows that at 0 min, the initial T[Al] and T[Mg] contents in molten steel were approximately 255 ppm and 5 ppm, respectively. After 30 min, T[Al] decreased significantly compared with the initial state, whereas T[Mg] increased substantially. A further comparison among slags with different MgO levels indicates that as the slag MgO content increased from 3 to 7 wt.%, the T[Al] content in steel fluctuated within a relatively narrow range and stabilized at approximately 100 ± 20 ppm. Meanwhile, T[Mg] exhibited only minor variation, remaining around 10 ± 1 ppm.

### 3.2. Effect of Slag–Steel Reaction on Non-Metallic Inclusions

#### 3.2.1. Typical Morphologies of Non-Metallic Inclusions

**Figure 3:**
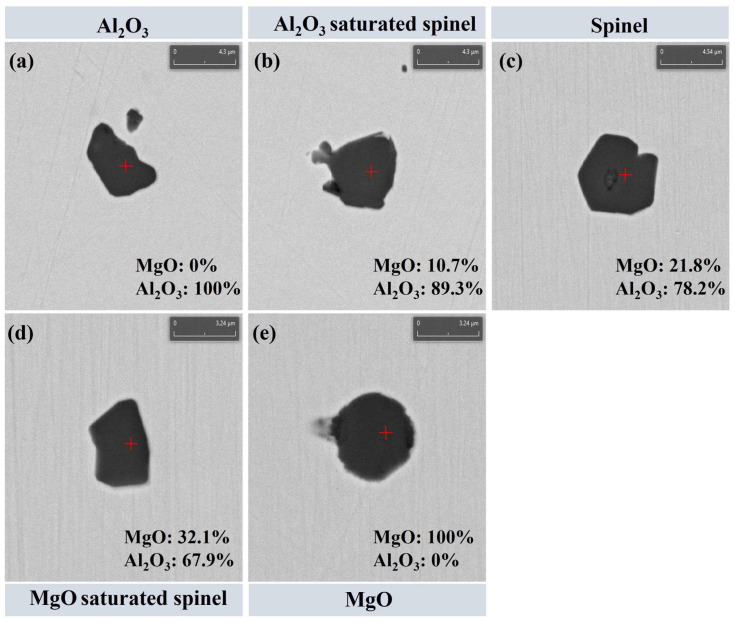
Typical morphology of inclusions: (**a**) Al_2_O_3_; (**b**) Al_2_O_3_ saturated spinel; (**c**) MgO·Al_2_O_3_; (**d**) MgO saturated spinel; (**e**) MgO.

In this work, only oxide inclusions formed and present in molten steel at high temperature are discussed. Secondary phases precipitated during solidification (e.g., MnS and TiN) are not considered, in order to eliminate the interference of solidification segregation and solid-state precipitation on the evolution behavior of high-temperature inclusions. Statistical results indicate that at 0 min of the slag–steel reaction, the oxide inclusions in steel were mainly composed of Al_2_O_3_ and MgO·Al_2_O_3_. After 30 min of reaction, the inclusion population shifted markedly toward MgO·Al_2_O_3_ and MgO, and these types accounted for more than 98% of the total inclusions. The remaining oxide inclusions contributed less than 2%, primarily consisting of minor CaO_x_(Al_2_O_3_)_y_ and CaO–MgO–Al_2_O_3_ complex inclusions. Therefore, to highlight the dominant inclusion types and their evolution, this study focuses on Mg–Al binary oxide inclusions. It should be noted that Ji et al. [26] reported a supersaturation phenomenon for MgO·Al_2_O_3_ inclusions, where the inclusion composition deviates within a certain range from the stoichiometric spinel point. Following this concept, the present study uses the MgO mass fraction as the criterion to further subdivide spinel-type inclusions into three categories: inclusions containing 19–29 wt.% MgO are defined as MgO·Al_2_O_3_; those with MgO < 19 wt.% are classified as Al_2_O_3_-saturated spinel; whereas inclusions with MgO > 29 wt.% are defined as MgO-saturated spinel. Accordingly, the Mg–Al binary oxide inclusions are categorized into five groups: (1) Al_2_O_3_; (2) Al_2_O_3_-saturated spinel; (3) MgO·Al_2_O_3_; (4) MgO-saturated spinel; and (5) MgO. Figure 3 presents the typical morphologies and corresponding compositional characteristics of these five Mg–Al binary oxide inclusion categories. As shown in Figure 3, Figure 3a corresponds to Al_2_O_3_ inclusions (MgO = 0 wt.%); Figure 3b shows Al_2_O_3_-saturated spinel inclusions (MgO = 10.7 wt.%); Figure 3c represents MgO·Al_2_O_3_ spinel inclusions (MgO = 21.8 wt.%); Figure 3d corresponds to MgO-saturated spinel inclusions (MgO = 32.1 wt.%); and Figure 3e shows MgO inclusions (MgO = 100 wt.%). These results demonstrate that the inclusion categories in this study can be unambiguously distinguished based on their MgO content, providing a robust basis for subsequent analyses of inclusion evolution and removal behavior.

#### 3.2.2. Types and Distributions of Non-Metallic Inclusions

**Figure 4:**
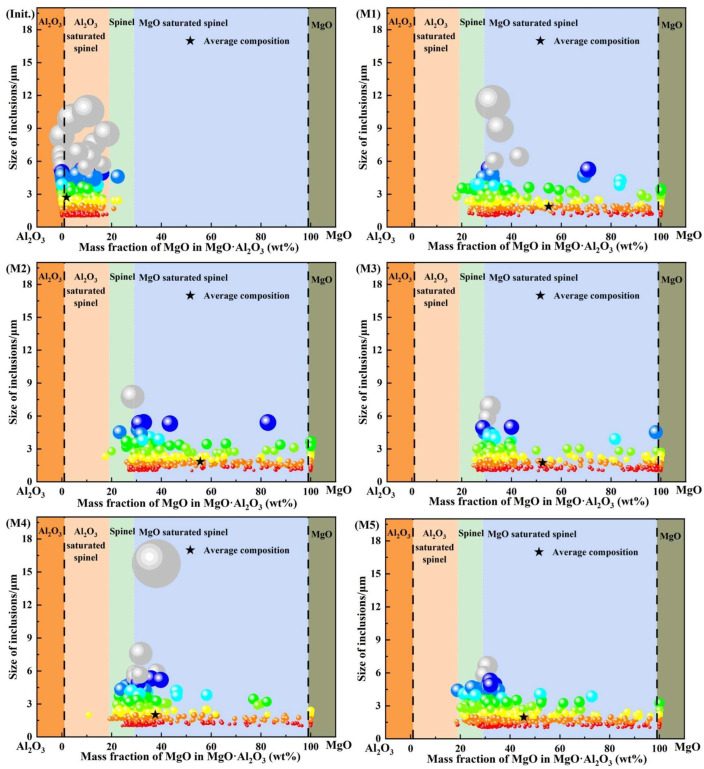
Compositions of major inclusions.

Figure 4 shows the compositional distributions of the dominant oxide inclusions at 0 and 30 min during the slag–steel reaction. In this plot, each sphere represents the projected position of an individual inclusion. The sphere diameter is positively correlated with the actual inclusion size, while the color scale is also linked to inclusion size, thereby reflecting the size distribution of inclusions. Based on the MgO mass-fraction range in Mg–Al binary oxide inclusions, inclusions were categorized into five groups and the corresponding compositional intervals were highlighted by colored regions.

As shown in Figure 4, at 0 min, the inclusions were mainly composed of Al_2_O_3_ and Al_2_O_3_-saturated spinel, with only a negligible fraction of MgO·Al_2_O_3_ inclusions. The mean inclusion composition fell within the Al_2_O_3_-saturated spinel region. In addition, the inclusions were generally fine, and the maximum size did not exceed 13 μm. After 30 min of reaction, the inclusion population underwent a pronounced compositional shift, becoming dominated by MgO-saturated spinel, accompanied by minor MgO·Al_2_O_3_ and MgO inclusions. The mean compositions of inclusions in all heats were located within the MgO-saturated spinel region. The inclusion size remained overall at a relatively low level: except for heat M4, where the maximum inclusion size reached approximately 16 μm, the maximum size in the other heats remained below 13 μm. A further comparison among different heats reveals that the mean inclusion compositions in heats M1–M3 were concentrated in the MgO-saturated spinel region corresponding to 50–60 wt.% MgO, whereas those in heats M4 and M5 tended to shift toward a lower MgO range of 40–50 wt.% within the same compositional domain.

**Figure 5:**
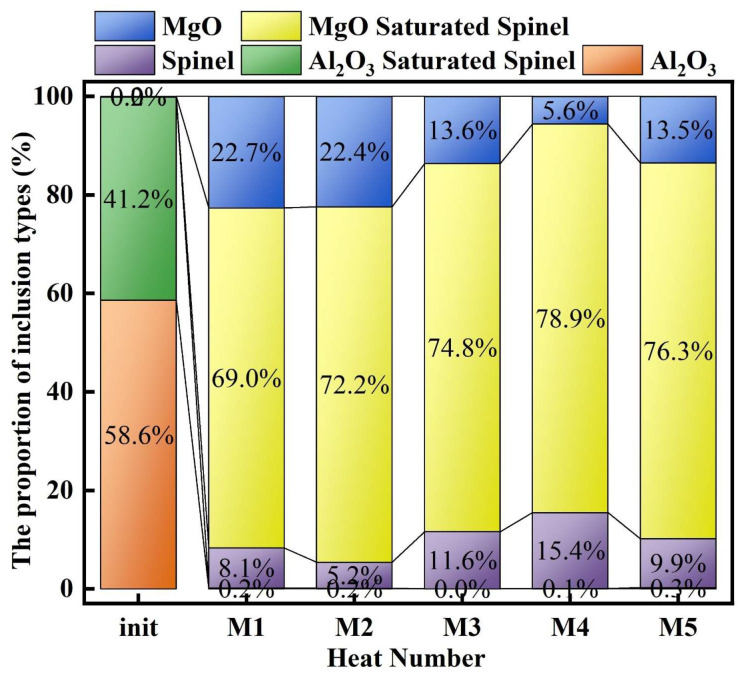
Proportion of inclusion types.

Figure 5 presents the proportion of different Mg–Al binary oxide inclusion types. As shown in Figure 5, at 0 min of the slag–steel reaction, the inclusions in molten steel were mainly composed of Al_2_O_3_ and Al_2_O_3_-saturated spinel, accounting for approximately 58.6% and 41.2%, respectively, whereas only a negligible fraction of MgO·Al_2_O_3_ inclusions was observed (∼0.2%). After 30 min of reaction, a pronounced transformation in inclusion types occurred. Al_2_O_3_ inclusions disappeared completely, and the proportion of Al_2_O_3_-saturated spinel decreased to below 0.5%. Consequently, the inclusion population became dominated by MgO-saturated spinel. With the initial MgO content in slag increasing from 3 to 7 wt.%, the fraction of MgO-saturated spinel exhibited an overall increasing trend (except for M5), reaching 69.0%, 72.2%, 74.8%, 78.9%, and 76.3% in heats M1–M5, respectively. In contrast, the proportion of MgO inclusions displayed an opposite tendency relative to MgO-saturated spinel. Except for M5 (MgO = 7 wt.%), the MgO fraction decreased overall with increasing slag MgO content, with values of 22.7%, 22.4%, 13.6%, 5.6%, and 13.5% for heats M1–M5, respectively. Meanwhile, the fraction of MgO·Al_2_O_3_ (spinel) inclusions fluctuated among different heats, accounting for 8.1%, 5.2%, 11.6%, 15.4%, and 9.9% in heats M1–M5, respectively.

#### 3.2.3. Inclusion Size

**Figure 6:**
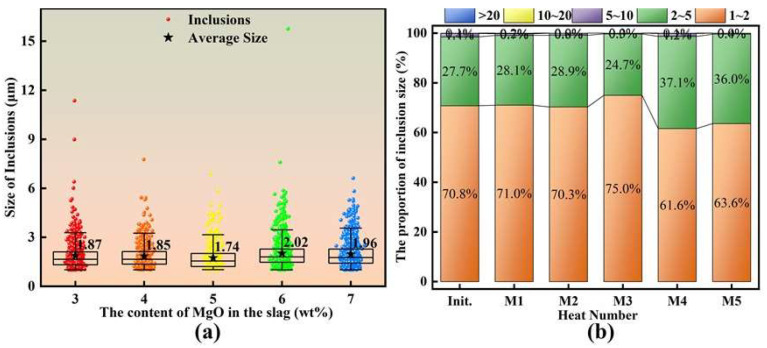
Average size and percentage size distribution of inclusions: (**a**) average size; (**b**) percentage size distribution.

Figure 6 presents the average inclusion size and the corresponding size distribution. As shown in Figure 6a, the projected size of each individual inclusion is represented by a sphere. With the initial MgO content in the designed slags increasing from 3 to 7 wt.%, the average inclusion size remained generally small and varied only slightly within the range of 1.8 ± 0.3 μm. Specifically, the average inclusion sizes in heats M1–M5 were 1.87 μm, 1.85 μm, 1.74 μm, 2.02 μm, and 1.96 μm, respectively.

Figure 6b further shows the fraction of inclusions in different size ranges at 0 and 30 min of the slag–steel reaction. The results indicate that inclusions were predominantly distributed in the 1–2 μm range, followed by the 2–5 μm range, whereas inclusions in other size intervals accounted for only a negligible portion, with a total fraction of less than 1%. More specifically, the fractions of 1–2 μm inclusions in heats M1–M5 were 71.0%, 71.3%, 75.0%, 61.6%, and 63.6%, respectively, while those of 2–5 μm inclusions were 28.1%, 28.9%, 24.7%, 37.1%, and 36.0%, respectively.

#### 3.2.4. Inclusion Content

**Figure 7:**
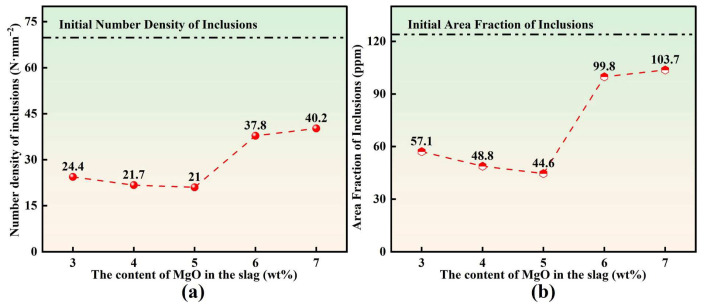
Inclusion content: (**a**) Number Density of inclusions; (**b**) Area fraction of inclusions.

At present, for inclusion statistics obtained using the automated inclusion scanning system Particle X, the inclusion number density (ND) and inclusion area fraction (AF) are commonly adopted as the primary indicators to evaluate inclusion content in molten steel. Figure 7 shows the variation in inclusion content during the slag–steel reaction as a function of slag MgO content. As shown in Figure 7a, the inclusion number density at 0 min was approximately 69.8 N·mm^−2^. After 30 min of reaction, inclusions floated up and were absorbed by slag, resulting in a pronounced decrease in number density. With the initial MgO content in slag increasing from 3 to 7 wt.%, the number density exhibited a clear “decrease–increase” pattern, with values of 24.4, 21.7, 21.0, 37.8, and 40.2 N·mm^−2^, respectively. The minimum ND was obtained at 5 wt.% MgO, which is consistent with the trend observed for the total oxygen content T[O] in molten steel. Figure 7b indicates that the inclusion area fraction at 0 min was approximately 124 ppm. After 30 min of reaction, AF decreased significantly by 16.4~64%, indicating that the inclusion content was effectively suppressed. Specifically, as the MgO content in the slag increased from 3 to 7 wt.%, AF first decreased and then increased, with corresponding values of 57.1, 48.8, 44.6, 99.8, and 103.7 ppm, respectively, reaching a minimum at 5 wt.% MgO. Overall, the evolution trend of AF is in good agreement with that of ND and T[O], further demonstrating that the MgO level in slag plays a significant regulatory role in inclusion removal efficiency and molten steel cleanliness.

## 4. Discussion

### 4.1. Effect of Refining Slag on Inclusions in Steel

**Figure 8:**
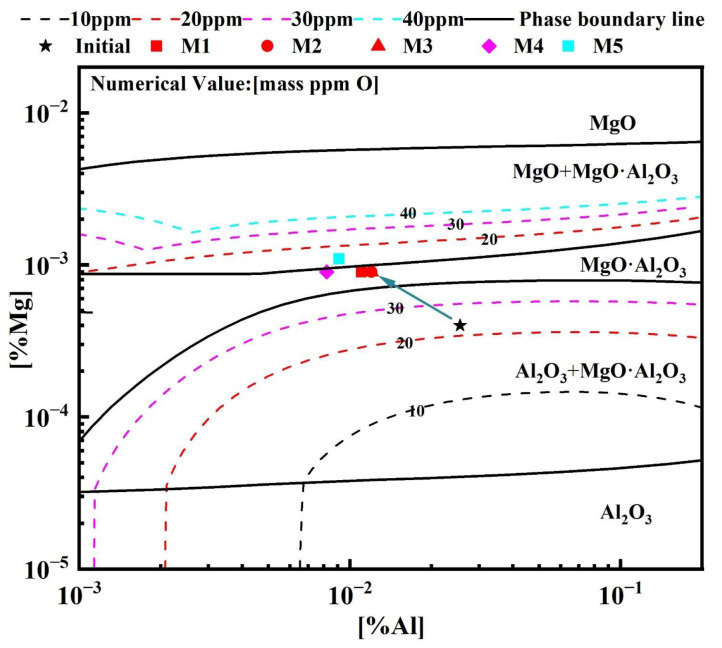
Mg–Al–O stability phase diagram of the experimental molten steel at 1600 °C.

To clarify the correspondence between the evolution of deoxidizing elements (Mg and Al) in molten steel and the compositional characteristics of oxide inclusions during the slag–steel reaction, the Mg–Al–O stability diagram at 1600 °C was calculated using FactSage 8.1. The dissolved oxygen content was set within the range of 10–40 ppm. Figure 8 presents the calculated Mg–Al–O stability diagram together with the projected representative inclusion compositions at different reaction stages. As shown in Figure 8, the representative inclusion composition at 0 min, indicated by the black pentagram, is located in the two-phase coexistence region of Al_2_O_3_ and MgO·Al_2_O_3_, which is consistent with the experimental observations. After 30 min of reaction, the Mg content in molten steel increased markedly while the Al content decreased, causing the inclusion composition points to shift toward the single-phase MgO·Al_2_O_3_ region. This trend suggests that the inclusion system gradually transformed from an initial Al_2_O_3_/spinel coexistence state to a thermodynamically more stable regime dominated by MgO·Al_2_O_3_. It is noteworthy that the composition point corresponding to heat M5 (slag MgO = 7 wt.%), represented by the blue square in Figure 8, falls into the MgO + MgO·Al_2_O_3_ two-phase region on the diagram. However, considering that the T[O] content after 30 min reached 41 ppm in this heat, the stable inclusion composition at this oxygen level should still lie within the single-phase MgO·Al_2_O_3_ region. Therefore, the small amount of MgO inclusions detected experimentally is more likely attributed to exogenous sources, such as entrainment from the slag itself [23,26] and/or dissolution of the MgO crucible at high temperature [40,41,42,43,44].

### 4.2. The Removal Rate of MgO·Al_2_O_3_ Inclusions by the Refining Slag Absorption

Compared with the state at 0 min, the total oxygen content T[O], as well as the inclusion number density and area fraction, decreased markedly after 30 min of slag–steel reaction, indicating that the slag–steel interaction effectively promotes inclusion removal. To quantitatively characterize the inclusion removal rate, previous studies [19,37] commonly introduced an apparent rate constant for inclusion removal, denoted as k_o_, as an evaluation parameter. In such kinetic models, the decrease in total oxygen content is treated as a macroscopic indicator of the reduction in oxide inclusions, i.e., it is assumed that the evolution of T[O] is predominantly governed by the formation and removal of oxide inclusions. Furthermore, by assuming that the deoxidation reaction follows first-order kinetics, the variation in total oxygen content with reaction time can be expressed as Equation (1):

(1) $$-\frac{{d[T[O]}_{t}]}{dt}=k_{o}(T{\left[O\right]}_{t}-T{\left[O\right]}_{e})$$

(2) $$-\ln(\frac{T{\left[O\right]}_{t}-T{\left[O\right]}_{e}}{T{\left[O\right]}_{0}-T{\left[O\right]}_{e}})=k_{o}t$$

In Equation (2), T[O]_t_ denotes the total oxygen content in molten steel at reaction time t, T[O]_e_ represents the equilibrium total oxygen content, T[O]_0_ is the initial total oxygen content at 0 min, and k_o_ is the apparent rate constant characterizing inclusion removal. By substituting the experimental data listed in Table 3 into Equation (2), the corresponding k_o_ values for different heats were obtained, and the results are also summarized in Table 3.

Previous studies have widely adopted the ratio of the chemical dissolution driving force ΔC (defined as the mass-fraction difference between slag components and the target inclusion phase at 1600 °C) to slag viscosity η, i.e., ΔC/η, as a key indicator for evaluating the capability of slag to remove specific inclusions [19,33,37,38,39]. It should be noted that the model employed in this study is applicable only to fully liquid slags at 1600 °C, namely those with a liquid fraction of 100% and without any solid precipitation. Therefore, reliable ΔC values cannot be calculated for partially molten slags containing solid phases, which defines the applicability and limitation of the present model. Based on the designed initial slag compositions in Table 2 and thermodynamic calculations using FactSage 8.1, the chemical driving force ΔC for the dissolution of magnesium aluminate spinel inclusions and the corresponding slag viscosity η at 1600 °C were determined for each slag system, and the results are listed in Table 3. It is noteworthy that slag M5 (MgO = 7 wt.%) corresponds to an MgO-saturated slag, in which solid phase precipitation occurs at 1600 °C. Consequently, M5 does not satisfy the fully liquid assumption required by the model, and its ΔC value cannot be obtained.

**Figure 9:**
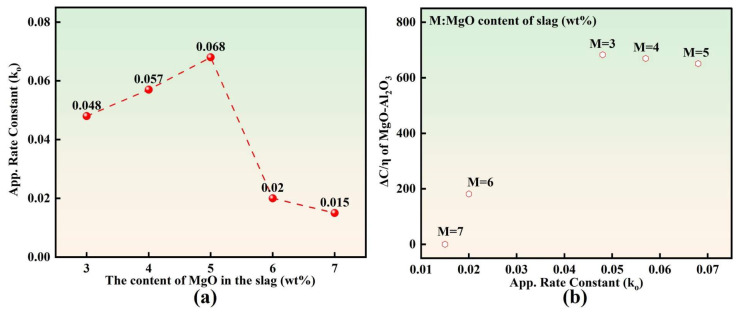
Relationship between ko and ΔC/η of inclusions: (**a**) ko; (**b**) ΔC/η of MgO·Al_2_O_3_ inclusions.

Figure 9 presents the relationship between the apparent inclusion removal rate constant k_o_ and ΔC/η for MgO·Al_2_O_3_ inclusions. As shown in Figure 9a, with the initial MgO content in slag increasing from 3 to 7 wt.%, the k_o_ value first increased and then decreased. This trend is opposite to that observed for the total oxygen content in molten steel. Figure 9b further demonstrates that a higher ΔC/η for MgO·Al_2_O_3_ inclusions corresponds to a larger k_o_, indicating that the slag exhibits a stronger capability to remove MgO·Al_2_O_3_ inclusions when the coupled parameter ΔC/η increases.

It should be noted that although MgO·Al_2_O_3_ is the dominant inclusion type in molten steel, small fractions of other oxide inclusions (e.g., MgO·Al_2_O_3_ and MgO) still exist in the system. Therefore, as an apparent kinetic parameter characterizing the overall inclusion removal process, k_o_ is not exclusively governed by ΔC/η for MgO·Al_2_O_3_ inclusions, but may also be influenced by the formation, transformation, and removal behaviors of other inclusion types. Consequently, the relationship between k_o_ and ΔC/η for MgO·Al_2_O_3_ inclusions is manifested as a positive correlation rather than a strictly exclusive dependence.

In this study, a self-developed computational code coupled with FactSage 8.1 was employed to systematically evaluate a series of refining slags with basicity *R* ranging from 4.0 to 8.0, MgO content varying from 0 to 7 wt.%, and a fixed Al_2_O_3_ content of 32 wt.%. Key parameters at 1600 °C, including the chemical dissolution driving force ΔC for MgO·Al_2_O_3_ inclusions, slag viscosity η, the coupled parameter ΔC/η, and the liquid fraction of slag, were obtained. In the calculations, ΔC and the slag liquid fraction were determined using the Equilib module with the FToxid database, whereas slag viscosity was calculated using the Viscosity module with the Melts database. Since viscosity calculations were performed only for fully liquid slags at 1600 °C, the calculated η values are equivalent to the effective viscosity η_e_ in this work.

**Figure 10:**
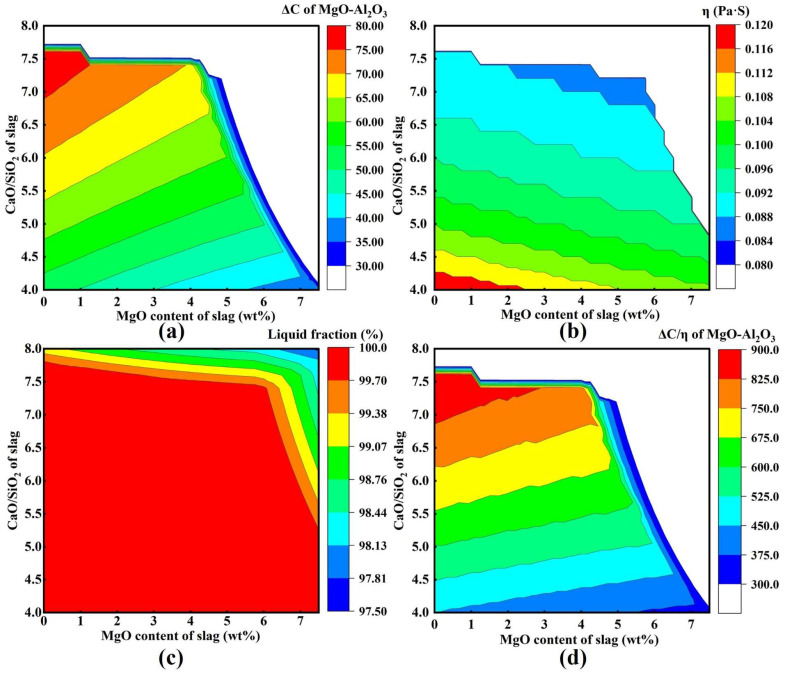
Adsorption capacity of different slags for MgO·Al_2_O_3_ inclusions at 1600 °C: (**a**) ΔC; (**b**) η; (**c**) Liquid Fraction; (**d**) ΔC/η.

Figure 10 summarizes the predicted adsorption capability of various slags toward MgO·Al_2_O_3_ inclusions at 1600 °C. As shown in Figure 10a, at a fixed basicity, ΔC decreases with increasing MgO content in slag, whereas at a constant MgO level, ΔC increases with increasing basicity R. Overall, ΔC reaches relatively high values in the regime of high basicity and low MgO content. Figure 10b indicates that at a given basicity, slag viscosity η decreases as MgO content increases; similarly, at a fixed MgO level, η also decreases with increasing basicity. In general, higher viscosities are predicted in slags with low basicity and low MgO content. Figure 10c shows that slags remain fully liquid at 1600 °C when R < 7.5 and MgO < 6 wt.%. As presented in Figure 10d, at a constant basicity, ΔC/η decreases with increasing MgO content, whereas at a fixed MgO level, ΔC/η increases with increasing basicity. Consequently, the highest ΔC/η values are obtained in the high-basicity and low-MgO region. From a theoretical perspective, refining slags with high basicity and relatively low MgO content, while maintaining a fully liquid state at 1600 °C, are more favorable for enhancing the adsorption and removal capability toward MgO·Al_2_O_3_ inclusions.

### 4.3. Adsorption Capacity of Refining Slag for Different Types of Inclusions

**Figure 11:**
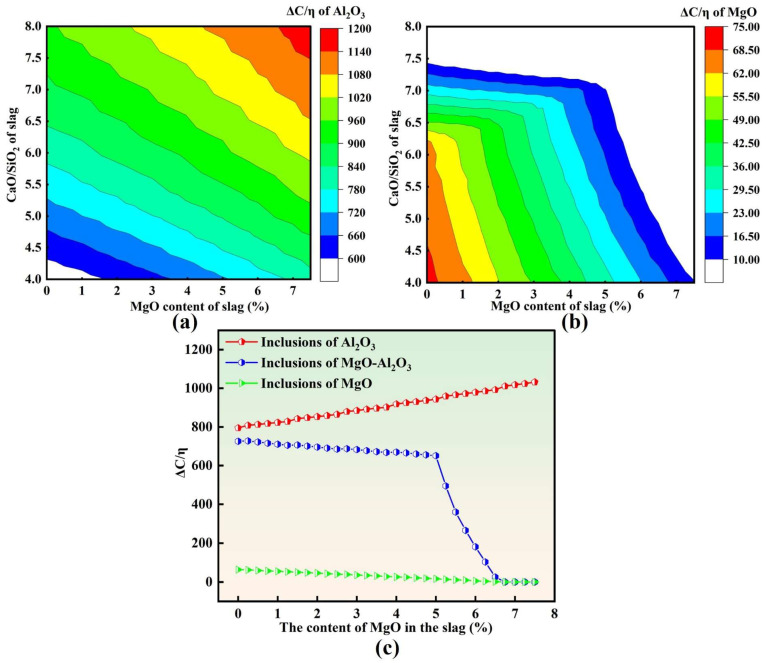
Adsorption capacity of slag for different types of inclusions: (**a**) Al_2_O_3_; (**b**) MgO; (**c**) different types.

Although MgO·Al_2_O_3_ is the dominant inclusion type in molten steel, small fractions of other oxide inclusions, such as Al_2_O_3_ and MgO, are also present. Given that slag exhibits markedly different adsorption and dissolution capabilities toward different inclusion types, it is necessary to further compare the ΔC/η characteristics associated with various inclusions. Figure 11 summarizes the predicted adsorption capability of slag toward different inclusion types.

As shown in Figure 11a, for Al_2_O_3_ inclusions, ΔC/η increases with increasing slag MgO content at a fixed basicity and also increases with increasing basicity R at a constant MgO level. Overall, the adsorption capability of slag toward Al_2_O_3_ inclusions is enhanced in the high-basicity and high-MgO region. In contrast, Figure 11b indicates that for MgO inclusions, ΔC/η decreases with increasing slag MgO content at a fixed basicity and likewise decreases with increasing basicity at a constant MgO level. Consequently, slag exhibits a relatively higher adsorption capability toward MgO inclusions in the low-basicity and low-MgO region, showing an opposite trend to that of Al_2_O_3_ inclusions.

Figure 11c further compares the variation in ΔC/η among different inclusion types as a function of slag MgO content. With the initial slag MgO content increasing from 0 to 7.5 wt.%, ΔC/η for Al_2_O_3_ inclusions increases continuously, whereas ΔC/η for both MgO·Al_2_O_3_ and MgO inclusions decreases. The magnitude of ΔC/η differs substantially among the three inclusion types, suggesting that, from a thermodynamic–kinetic perspective, slag has a stronger adsorption capability toward Al_2_O_3_ inclusions than toward MgO·Al_2_O_3_ inclusions, while MgO inclusions exhibit the weakest adsorption capability for slag absorption.

When these calculations are combined with the experimental observations, a consistent trend emerges. For slags with MgO ≤ 5 wt.%, increasing slag MgO content leads to a pronounced rise in ΔC/η for Al_2_O_3_, whereas the decrease in ΔC/η for MgO·Al_2_O_3_ remains relatively limited. As a result, the difference between ΔC/η (Al_2_O_3_) and ΔC/η (MgO·Al_2_O_3_) gradually expands. This implies that, within the MgO ≤ 5 wt.% range, the slag’s capability to remove MgO·Al_2_O_3_ inclusions remains relatively stable, while its removal efficiency for Al_2_O_3_ inclusions is significantly enhanced. This provides a mechanistic explanation for the experimental finding that T[O], inclusion number density, and area fraction continuously decreased with increasing slag MgO content (up to 5 wt.%), reaching the minimum values in heat M3 (MgO = 5 wt.%).

Notably, when the slag MgO content exceeds 5 wt.%, ΔC/η for MgO·Al_2_O_3_ inclusions exhibits a pronounced cliff-like decline, indicating a sharp deterioration in the slag’s adsorption and removal capability toward spinel inclusions. This behavior is consistent with the significantly higher T[O], inclusion number density, and area fraction observed in heats M4 and M5 compared with heats M1–M3. These results collectively suggest that an optimal MgO window exists in LF refining slag: a moderate increase in MgO (≤5 wt.%) is beneficial for inclusion removal, whereas excessively high MgO content can substantially weaken the absorption of spinel inclusions and ultimately deteriorate molten steel cleanliness.

**Figure 12:**
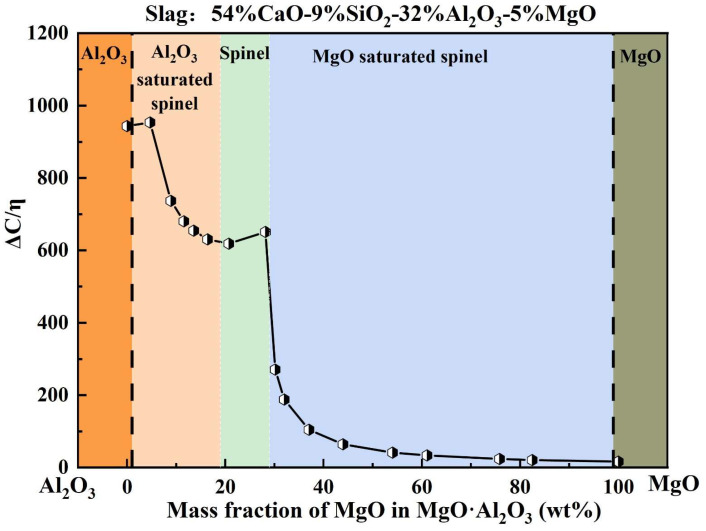
Adsorption capacity of slag for Mg–Al binary inclusions with different MgO contents.

During high-temperature refining, the steel–slag–refractory system involves multiple coupled reactions, including inclusion modification, interfacial adsorption, and refractory erosion. Moreover, slag MgO content often increases further due to refractory dissolution, which significantly complicates the evolution and removal behavior of inclusions. To quantitatively evaluate the slag adsorption capability toward Mg–Al binary inclusions with different MgO fractions, Figure 12 presents the calculated dependence of slag adsorption capability on inclusion composition.

As shown in Figure 12, heat M3 (54% CaO–9% SiO_2_–32% Al_2_O_3_–5% MgO) was selected as a representative slag system. The variation in ΔC/η was calculated for Mg–Al binary inclusions with different MgO contents to simulate the dynamic response of slag adsorption capability during inclusion modification driven by Mg enrichment, i.e., the progressive transformation of inclusions from Al_2_O_3_-rich compositions to MgO-rich spinel-type compositions. The results show that when the MgO content in inclusions is within 0–5 wt.%, ΔC/η remains at approximately 950 ± 20. As the inclusion MgO content increases to 5–28.2 wt.%, ΔC/η decreases to around 700 ± 50. Notably, once the inclusion MgO content exceeds 29 wt.%, ΔC/η drops sharply to approximately 150 ± 150, exhibiting a pronounced decline. This segmented behavior suggests that, from a theoretical perspective, the slag possesses a strong adsorption/removal capability for Al_2_O_3_, Al_2_O_3_-saturated spinel, and MgO·Al_2_O_3_ inclusions, whereas its adsorption/removal capability toward MgO-saturated spinel and MgO inclusions is substantially weaker. Accordingly, when inclusions become excessively enriched in MgO, they are expected to be increasingly difficult for slag to capture and remove effectively.

Based on these findings, it is recommended to control the MgO content in Mg–Al binary oxide inclusions below 29 wt.% to avoid entering the regime where slag adsorption capability deteriorates sharply, thereby facilitating improved cleanliness control in bearing steel production.

## 5. Conclusions

This study systematically investigated the effects of the initial MgO content (3–7 wt.%) in LF refining slag on steel cleanliness and inclusion removal behavior in bearing steel through laboratory-scale slag–steel reaction experiments. By correlating the evolution of molten steel chemistry with the characteristics of oxide inclusions, the following conclusions can be drawn:

1. Slag–steel reaction significantly promotes inclusion removal. After the reaction, the total oxygen content T[O], inclusion number density, and inclusion area fraction decreased markedly. With slag MgO content increasing from 3 to 7 wt.%, these three cleanliness indicators exhibited a consistent “decrease–increase” trend, reaching minimum values at 5 wt.% MgO. This demonstrates the existence of an optimal MgO window in refining slag for cleanliness control.
2. The apparent inclusion-removal rate constant k_o_ is positively correlated with ΔC/η for MgO·Al_2_O_3_ inclusions. Here, ΔC/η represents the ratio of the chemical dissolution driving force ΔC to slag viscosity η at 1600 °C. This correlation indicates that inclusion removal efficiency is jointly governed by thermodynamic driving forces and slag transport properties.
3. Slag adsorption capability toward Mg–Al binary inclusions decreases with increasing inclusion MgO content. Notably, when the MgO content in inclusions exceeds 29 wt.%, the adsorption capacity undergoes an abrupt drop, indicating a pronounced cliff-like attenuation behavior. Therefore, to maintain high inclusion removability and improve cleanliness control, the MgO content in Mg–Al binary oxide inclusions should preferably be kept below 29 wt.%.

## Figures and Tables

**Table 1:** Chemical Composition of Experimental Steel Grade, mass%.

C	Si	Mn	P	S	Al	Cr	Mg	N	O
0.95	0.28	0.32	0.0075	0.014	0.0255	1.51	0.0005	0.0027	0.0058

**Table 2:** Chemical composition of refining slag mass%.

Heat No.	Basicity (CaO/SiO_2_)	Content (Mass%)				
		CaO	SiO_2_	MgO	Al_2_O_3_	MgO Saturation
M1	6	55.7	9.3	3	32	6.2
M2	6	54.9	9.1	4	32	6.3
M3	6	54.0	9.0	5	32	6.4
M4	6	53.1	8.9	6	32	6.6
M5	6	52.3	8.7	7	32	6.7

**Table 3:** Data related to the removal efficiency of MgO·Al_2_O_3_ inclusions.

Heat No.	Total Oxygen (ppm)			k_o_	η at 1600 °C (Pa·S)	△C of MgO·Al_2_O_3_ (Mass %)
	30 min	Equilibrium	Initial			
M1	19	7.0	58	0.048	0.093	63.5
M2	17	7.8		0.057	0.092	61.6
M3	15	8.6		0.068	0.092	59.9
M4	36	9.4		0.020	0.091	16.5
M5	41	10.1		0.015	0.090	--

## Data Availability

The original contributions presented in this study are included in the article. Further inquiries can be directed to the corresponding author.
